# A Development and Comparison Study of PVDF Membranes Enriched by Metal–Organic Frameworks

**DOI:** 10.3390/polym17091140

**Published:** 2025-04-22

**Authors:** Tatiana Pisarenko, Nikola Papež, Mohammed A. Al-Anber, Rashid Dallaev, Klára Částková, Ştefan Ţălu

**Affiliations:** 1 Department of Physics, Faculty of Electrical Engineering and Communication, Brno University of Technology, Technická 2848/8, 61600 Brno, the Czech Republic; tatiana.pisarenko@vut.cz (T.P.); papez@vut.cz (N.P.); rashid.dallaev@vut.cz (R.D.); 2 Department of Chemistry, Faculty of Sciences, Applied Science Private University, P.O. Box 166, Amman 11931, Jordan; masachem@mutah.edu.jo; 3 Central European Institute of Technology, Purkyňova 656/123, 61200 Brno, the Czech Republic; klara.castkova@ceitec.vutbr.cz; 4 Department of Ceramics and Polymers, Faculty of Mechanical Engineering, Brno University of Technology, Technická 2896/2, 61600 Brno, the Czech Republic; 5 Directorate of Research, Development and Innovation Management (DMCDI), Technical University of Cluj-Napoca, Constantin Daicoviciu Street, No. 15, 400020 Cluj-Napoca, Cluj County, Romania

**Keywords:** contact angle, dye adsorption, electrospinning, membranes, metal-organic frameworks, methylene blue, nanofibers, photoluminescence, polyvinylidene fluoride

## Abstract

This study is concerned with the research and development of nanofibrous hybrid materials functioning as membranes composed of polyvinylidene fluoride (PVDF) polymer and enriched with metal–organic frameworks (MOFs) as dopants for the adsorption and detection of gases, dyes, and heavy metals in wastewater. Several types of nanofiber composites are fabricated by electrostatic spinning. The prepared samples and their chemical, optical, and material properties are analyzed. Subsequently, the preliminary investigation of dye removal is conducted. Accordingly, the design and investigation of these nanofibrous structures may contribute to addressing critical environmental and technological challenges.

## 1. Introduction

The polyvinylidene fluoride (PVDF) and metal–organic frameworks (MOFs) examined in this paper are two material classes that have gained considerable importance in recent decades in the context of advanced technologies such as filtration, catalysis, and gas adsorption. Their potential lies not only in their individual physicochemical properties but also in the synergies that arise when they are combined. This paper explores in detail the interactions between PVDF and MOFs, with an emphasis on their synergistic properties, which may lead to new opportunities for the design of high-performance materials [1].

PVDF, chemical formula (C2H2F2)n, is a semi-crystalline fluorinated polymer whose unique properties are based on its chemical structure, which is made up of linear chains containing moderately strong dipole interactions between fluorine and carbon. These interactions give PVDF high stability against aggressive chemicals, high-temperature resistance, and mechanical strength. PVDF is also a promising material for advanced technologies, including energy harvesting devices, sensors, and actuators, owing to its piezoelectric, ferroelectric, and dielectric properties. However, despite its advantages, several challenges hinder its broader application in electronics [2]. One major issue is the control over its crystalline phase, particularly the electroactive β-phase, which is critical for optimal performance but difficult to achieve uniformly during fabrication [3]. Additionally, PVDF membranes often face limitations in mechanical flexibility and structural integrity, especially when subjected to repeated bending or thermal cycling in wearable or flexible electronics [4]. Long-term stability and environmental resistance, such as performance degradation under humidity or UV exposure, also present significant obstacles [5]. Moreover, integrating PVDF membranes with other components in electronic circuits remains complex due to surface compatibility and processing constraints [6]. These challenges require continuous material innovations, like integrating metal–organic frameworks (MOFs), to enhance performance and reliability in practical applications. If attention is focused on the area of filtration and separation, its ability to form microporous structures, particularly in the case of membranes, makes it an ideal material for this purpose, such as in desalination, water purification processes, and dye removal applications. However, traditional PVDF membranes may have limitations in terms of their dye adsorption capacity and selectivity for specific dyes.

MOFs are a class of porous materials that consist of metal ions or clusters coordinated with organic ligands. This type of material is characterized by its exceptional porosity, high surface area (over 1000 m^2^/g), and specific adsorption capacity. MOFs exhibit high flexibility in synthesis, which allows changing their topology, pore size, and chemical functionality through modification of metal cores and organic ligands. This leads to extraordinary specificity in the selective adsorption of gases such as CO2, CH4, and H4, making them ideal for applications in gas storage and separation, catalysis, sensors, and also for use in environmental technologies such as harvesting carbon from air or remediation of water polluted by textile dyes and heavy metal [7].

Combining PVDF and MOFs into hybrid materials allows the synergy between the mentioned properties of polymer and porous materials to be exploited. The implementation of MOFs into the PVDF matrix can significantly improve the separation and adsorption properties of polymeric materials. This integration leads to materials with specific pore structures and improved selective adsorption capabilities, while PVDF provides high mechanical stability and material durability in aggressive environments. For example, Chen et al. used MOF-808@PVDF microspheres for recovering phosphorus from wastewater and waste-activated sludge [8], Eze et al. added UiO-66 and ZIF-8 in PVDF as selective membranes for lithium-ion separation [9], Muhtar et al. prepared electrospun PAN/PVDF nanofibers for dye filtration, which were tested with methylene blue (MB) and crystal violet (CV) [10]. These represent a selection of current and advanced experiments in environmental remediation; thus, the contribution of new findings and explorations remains essential.

Among the many applications of MOF@PVDF hybrid materials, dye removal from wastewater is particularly compelling due to the increasing environmental burden of textile effluents. Traditional PVDF membranes, while robust and chemically stable, often suffer from limited adsorption capacity and lack specificity toward organic pollutants. The incorporation of MOFs, owing to their exceptionally high surface areas, functionalized pore structures, and tunable chemistry, offers a transformative pathway to address these limitations. By combining mechanical stability of PVDF with MOFs’ targeted sorption properties, these hybrid membranes represent a promising advancement in dye removal technologies, potentially outperforming conventional systems in both capacity and selectivity [11].

In electrochemical applications such as supercapacitors or batteries, MOFs can improve the ion adsorption capacity of materials and electrochemical conductivity [12]. In the case of hydrogen technologies, which focus on efficient storage and distribution of hydrogen gas, MOFs combined with PVDF can create highly efficient materials for hydrogen sorption and separation [13,14,15]. Due to the high surface area of MOFs and the electrochemical properties of PVDF, it is possible to develop hybrid materials with high specific capacitance for hydrogen technologies, opening new opportunities for the development of hydrogen fuel cells with higher energy efficiency.

Although the combination of PVDF and MOFs represents promising directions for the development of new materials, there are also challenges that need to be addressed to realize their full potential. One of the main challenges is to ensure sufficient dispersion of MOFs in the PVDF matrix and to ensure their long-term stability when used under realistic conditions. Another problem is optimizing the mechanical properties, as the addition of MOFs can affect the elasticity and strength of the resulting composite. The selection of a suitable synthetic method to allow reproducibility and control of the structure of the hybrid materials is also an important factor to be solved.

## 2. Material and Methods

### 2.1. Solution Synthesis and Its Components

The base material used for the membrane was PVDF (Sigma Aldrich, St. Louis, MO, USA) with a molecular weight of 275,000 g/mol.

The solution was further supplemented with four commercially purchased MOFs: iron-based Basolite F300 (Sigma Aldrich, St. Louis, MO, USA), also known as Fe-BTC or Iron 1,3,5-benzenetricarboxylate, trimesic acid ligand, formula C9H3FeO6; zinc-based Basolite Z1200 (Sigma Aldrich, St. Louis, MO, USA), also known as ZIF-8 or 2-Methylimidazole zinc salt, 2,2′-bipyridine ligand, formula C8H10N4Zn; zirconium-based Universitetet i Oslo-66 (Nanochemazone, Leduc, AB, CA), also known as UiO-66 (Zr) or Zirconium 1,4-dicarboxybenzene, terephthalic acid ligand, formula C48H28O32Zr6; and chromium-based Materials of Institute Lavoisier 101 (Nanochemazone, Leduc, AB, CA), also known as MIL-101 (Cr), terephthalic acid ligand, formula C18H6Cr3O12.

The PVDF granulate, together with MOF powders, was dispersed and mixed in a dimethylformamide and acetone (DMF/Ac) solvent in a 7:3 ratio. Subsequently, the solution was heated for 24 h on a stirrer at 80 °C and 200 rpm. All four types of solutions were mixed in a ratio of 15% PVDF and 8% MOF. These solutions and subsequently the specimens were labeled as Fe-MOF@PVDF, Zn-MOF@PVDF, Zr-MOF@PVDF, and Cr-MOF@PVDF. In the paper, they are often distinguished by red, blue, green, and orange colors, respectively, for better clarification.

### 2.2. Fabrication of Nanofibrous Membranes

The membrane was formed from composite nanofibers produced by electrostatic spinning. An 4SPIN instrument (Contipro, Dolní Dobrouč, the Czech Republic) was used in a configuration where a single needle was used as the emitter from which the solution flowed and a collector in the form of a rotating cylinder on which the nanofibers were deposited. The cylinder was covered with aluminum foil approximately the size of A4 paper. This allowed the nanofiber mats to be easily handled and removed from the rotating cylinder. The parameters for all four materials were identical: high-voltage 50 kV, emitter/collector distance 20 cm, collector speed 2000 rpm, emitter flow rate 23 μL/min, needle size 17 GA, syringe volume 10 mL, ambient temperature 24 °C, and humidity 21 °C. After electrospinning, the nanofiber mat was placed in a dryer at 80 °C for one hour.

### 2.3. Instruments and Procedures Used for Analysis

Fourier transform-infrared spectroscopy (FTIR) in absorbance mode by Vertex 70s (Bruker, Billerica, MA, USA) provided an accurate fingerprint of the individual samples. After measurement, a baseline correction was applied to adjust the absorbance spectra. The most dominant region of the spectrum in the range of 1800 to 400 cm^−1^ was shown.

The photoluminescence (PL) spectra of membranes were acquired at room temperature using a 355 nm laser with 3.5 mW power by the Alpha300R confocal Raman imaging system (Witec, Ulm, Germany). The integration time was 10 s, and the number of accumulations was 25. The spectrum was measured in the range of 350 to 800 nm using 40× magnification objective.

The condition and quality of the formed membranes were examined using the LYRA3 (Tescan, Brno, the Czech Republic) scanning electron microscope (SEM) with a backscattered electron (BSE) detector and the X-Max 50 EDS detector (Oxford Instruments, Oxford, UK) for elemental analysis. The BSE detector was used to verify the presence of MOFs in the fibers, which had higher contrast with heavier elements. The EDS detector was used to confirm and investigate the amount of the elements in the material. For regular imaging, an accelerating voltage of 2 kV was used. When using a BSE or EDS detector, the voltage was increased to 10 kV for better material contrast and element detection.

The contact angle and wettability of the samples were measured using the See System (Advex Instruments, Brno, the Czech Republic). Demineralized water of 3 μL was applied on the sample surface. Ten measurements were taken from several places on each sample, and the result averaged for possible random and systematic errors. After the application of the droplet, it was held for 5 s, and then the image was captured, and the contact angle was calculated.

**Figure 1:**
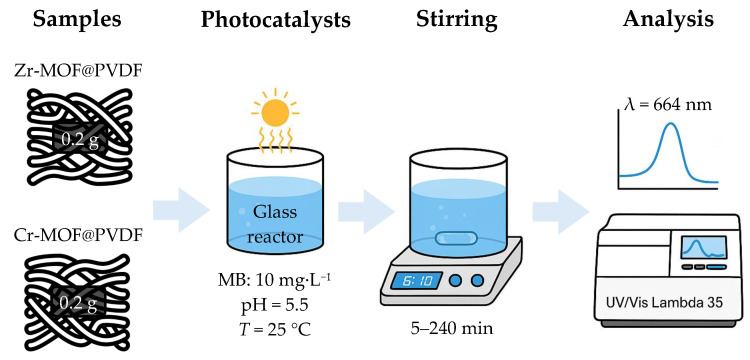
Summary diagram of described photocatalyst process.

The photodegradation of 95% methylene blue (Sigma Aldrich, St. Louis, MO, USA) was conducted using a glass reactor containing the MB solution (10 mg L^−1^) with a controlled pH = 5.5 and *T* = 25 °C (±1 °C). The photocatalyst (e.g., Zr-MOF@PVDF and Cr-MOF@PVDF) was immobilized on a glass substrate, wherein 0.2 g of Zr-MOF@PVDF or Cr-MOF@PVDF were used. The system was stirred continuously within 5 to 240 min, and samples were taken regularly to monitor degradation using UV–Vis spectroscopy at λ = 664 nm. The degradation efficiency was calculated. Blank tests were performed for comparison, and reusability studies assessed the catalyst’s stability over multiple cycles. The optical absorbance spectra of MB were analyzed using a UV–Vis Lambda 35 spectrophotometer (PerkinElmer, Waltham, MA, USA). A basic diagram of the overall photocatalysis process is shown in Figure 1.

## 3. Results and Discussion

On the basis of the parameters and procedures mentioned in the previous section, MOF-doped PVDF nanofibers based on iron, zinc, zirconium, and chromium were developed according to previous experimental reports [16]. The fabrication was carried out without any complications that could significantly affect the defectivity of the fibers.

### 3.1. Spectral Analysis of Fabricated Material

**Figure 2:**
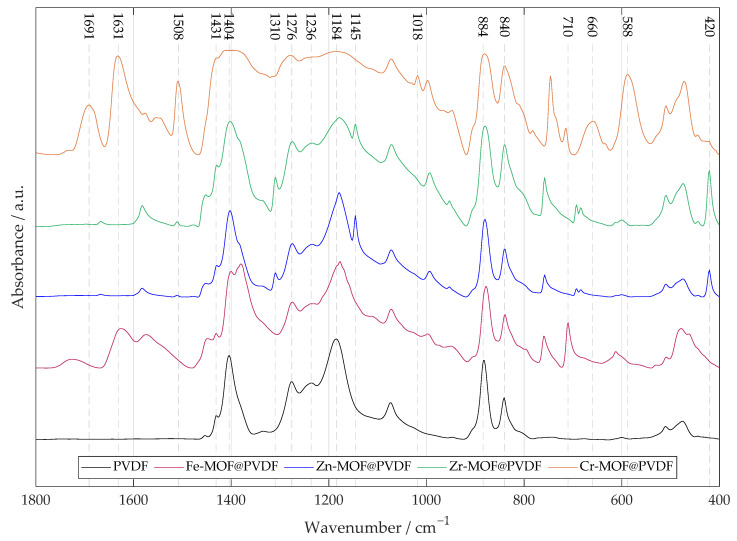
The FTIR of four types of PVDF nanofibrous membranes with MOF based on iron, zinc, zirconium, and chromium. A pure PVDF fiber membrane is added for reference. For MOF-based materials, metal–oxygen bonds are typically found at lower values of ∼800 to 600 cm^−1^. Other several important peaks are marked in the spectrum indicating the specific vibrational–rotational motion described in the paper.

Polyvinylidene fluoride is a polymer with a complex FTIR spectrum that varies depending on the crystalline phase (most interesting phases are α, β, γ, δ). In the case of a composite material where MOF is added to PVDF, it is useful to provide the plain PVDF nanofibers as a reference to help distinguish the peaks of the other samples, as can be seen in Figure 2.

The α-phase has a dihedral polymer chains conformation (TGTG′) in a non-polar structure. β-phase is characterized by an ordered conformation (TTTT), which contributes to its electroactivity. The alignment of fluorine atoms creates a significant dipole moment, as fluorine is highly electronegative. γ-phase consists of the T3GT3G′ conformation. Both β- and γ- are polar phases. The β-phase is represented by the CF_2_ rocking peak at 840 cm^−1^, 1431 cm^−1^, and by the CF2 stretching peak at 1276 cm^−1^ [17,18]. Combination of α-, β- and γ-phases shows peaks at 884 cm^−1^ and 1404 cm^−1^ [17]. For the γ-phase, a smaller CF2 stretching peak at 1236 cm^−1^ and CF_2_ symmetric stretching peak at 1184 cm^−1^ are characteristic, indicating a combination of γ- and β-phase [17].

The sharp peak at 710cm^−1^ for Fe-MOF@PVDF represents the Fe–O stretching vibrations. Band at 1625 cm^−1^ is assigned to benzene ring vibrations [19].

The Zn-MOF@PVDF and Zr-MOF@PVDF spectra are quite similar and differ more in the ratio of peak intensities than in their positions. The strong values at 420 cm^−1^ show the stretching vibration of metal–N [20,21]. Compared to the other samples, they show the peaks at 1145 cm^−1^ or 1310 cm^−1^ assigned to aromatic stretching mode (C–N) in the imidazole groups, which confirms good incorporation in polymer matrix and retains the original chemical structure [22].

The peaks observed at 1691, 1631 and 1402 cm^−1^ in the FTIR spectrum of Cr-MOF@PVDF are primarily attributed to the asymmetric and symmetric stretching vibrations of carboxylate groups (O–C–O) derived from the Cr-MOF terephthalic acid linker coordinated to chromium clusters. However, the value ∼1402 cm^−1^ is overlapped by another peak defining deformation vibration of CF2 groups in PVDF. At the same time, its position and intensity can also provide insights into the crystalline phase, particularly the β-phase, known for its piezoelectric properties. MOFs often exhibit broad and unsharp bands in the FTIR spectra, which can lead to overlapping signals, and for Cr-MOFs in particular, this misalignment is noticeable as can be seen. The value of 1508 cm^−1^ is assigned to stretching of the aromatic ring (C–C) bonds. The peak at 1018 cm^−1^ is attributed to the in- and out-of-plane bending of the (C–H) bond. Values 660 cm^−1^ and 588 cm^−1^ are related to stretching vibrations of oxygen (Cr–O) [23,24].

The slight shift between values may be due to differences in crystallinity, orientation of polymer chains, or interactions with other components in the composite.

**Figure 3:**
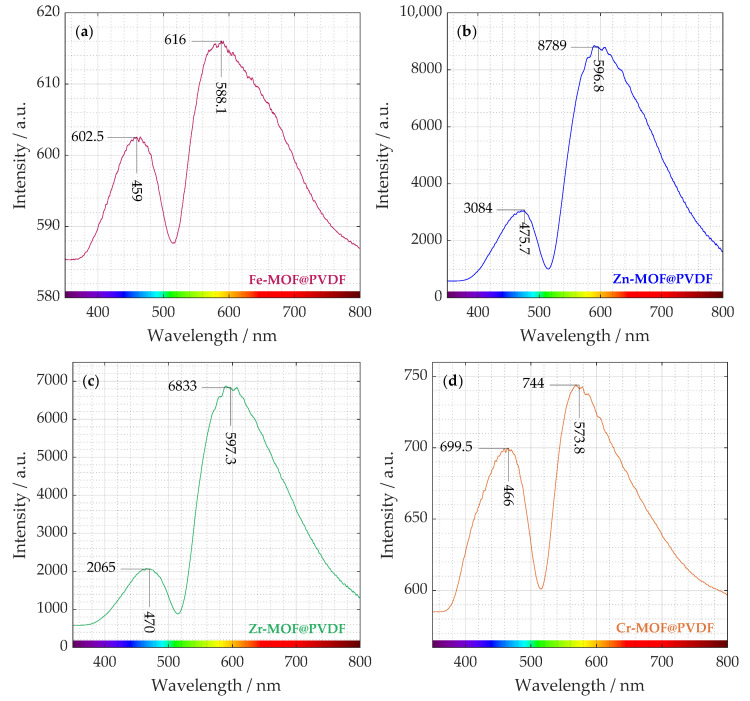
Measured photoluminescence spectra distributed in four plots. Different intensities can be distinguished from each curve of (**a**) iron-based, (**b**) zinc-based, (**c**) zirconium-based, and (**d**) chromium-based MOF incorporated in PVDF nanofibers.

By analyzing the emitted light, photoluminescence (PL) spectroscopy provides insights into the processes and pathways involved in the recombination of photogenerated electron-hole pairs. The photoluminescence analysis in Figure 3 was divided into individual spectra separately due to the resolution of their intensities, which varied significantly from material to material.

The presence of two distinct peaks suggests multiple radiative recombination pathways, possibly from different defect states or charge carrier trapping. The organic ligands within the MOF structure also can contribute to photoluminescence through π → π* or *n* → π* transitions. As can be seen, the intensities of these transitions are different. Zn-MOF@PVDF and Zr-MOF@PVDF have a multiple lower intensity in the ∼470 nm region than in the ∼600 nm region. Fe-MOF@PVDFs and Cr-MOF@PVDFs also show higher photoluminescence intensity at ∼580 nm but not in such a degree [25].

Similar weak fluorescence values at the maximum emission peak at 455 nm as in the case of our experiment were measured by Wu. Z. et al. on Fe-MOF. In Figure 3a, Fe-MOF@PVDF is the sample with the lowest intensity (602.5 and 616). This indicates that the fluorescence quenching of Fe-MOF is caused by the internal filtration effect (IFE) and energy transfer [26].

It is evident that Zn-MOF@PVDF and Zr-MOF@PVDF samples exhibit up to an order of magnitude higher intensity than Fe-MOF@PVDF and Cr-MOF@PVDF. The observed PL dominance suggests that oxygen vacancies and defect sites play a crucial role in the structure and recombination processes of the material. It is well established that oxygen vacancies and defects within the material act as binding sites for electrons, promoting the creation of excitons. These excitons, in turn, generate new energy levels that sit close to the bottom of the conduction band. As the number of oxygen vacancies and defects increases, so does the population of excitons, ultimately leading to a stronger PL intensity [27].

In Figure 3b, a weaker emission appears in the blue region (around ∼470 nm). This could be attributed to intrinsic defect-related emissions, ligand-to-metal charge transfer (LMCT), or surface trap states in Zn-MOF. For instance, studies have shown that electrospun fibers containing rhodamine B encapsulated within ZIF-71 (a type of Zn-MOF) dispersed in a PVDF matrix exhibit tunable luminescence with a quantum yield exceeding 90% [28]. Much stronger emission occurs in the near red region (∼600 nm). This peak suggests electronic transitions within the Zn-MOF structure, possibly due to ligand-centered fluorescence or Zn2+-induced luminescence. It could also indicate energy transfer between the MOF and PVDF matrix, leading to red-shifted emission.

The interaction between Zn2+ and the organic ligands can lead to metal-to-ligand charge transfer (MLCT) or ligand-to-metal charge transfer, contributing to the overall luminescence. A study on fluorescent Zn(II)-based MOFs highlighted that luminescence can originate from the organic linker and metal node, where the ligand acts as a rigid structural component and simultaneously provides the emissive character of the structure [29].

In Figure 3d, the emission peak near 570 nm can be associated with *d* → *d* transitions within the chromium centers. Chromium is a transition metal, and its ions have electrons in the *d*-orbitals. These transitions involve electron excitations between split *d*-orbitals of the metal ion and are influenced by the ligand field environment. In chromium-based MOFs, such *d* → *d* transitions can result in emissions in the visible to near-infrared regions [30]. The observed emission around 400 to 500 nm in spectrum likely corresponds to LMCT processes. On the other hand, for Zr-MOF@PVDF, Zr(IV) is highly electron-deficient with no *d* → *d* electronic transitions [31]. *d* → *d* transitions, which are electronic transitions between *d*-orbitals, are a key source of color and light absorption/emission in many transition metal compounds. That is why UiO-66 is typically white. Although these transitions are absent, the luminescence in UiO-66 may still occur, but it will have a different origin. Possible sources of Zr-MOF@PVDF photoluminescence include ligand-based luminescence or defect-related luminescence similarly, as with Zn-MOF@PVDF.

**Figure 4:**
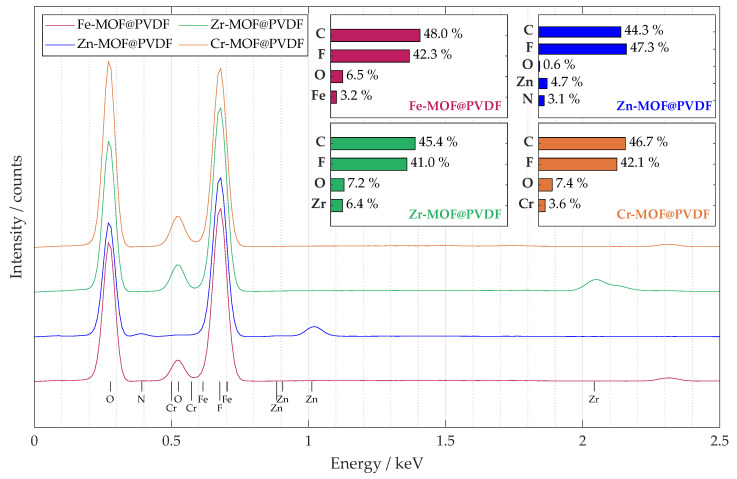
The captured EDS spectrum from the 250 μm × 250 μm map shows the average elemental distribution of the studied samples. A bar chart with the percentage distribution of the main elements is also provided for each material.

A summary EDS spectrum in Figure 4 confirmed the presence and content of all the important elements expected in the nanofiber material. The spectrum was obtained from a sample map of sufficient size 250μm × 250 μm. The accumulation from such a large area provided that the MOFs would be detected with high confidence, and their distribution is more uniform. The curves for all four materials studied are shown in the plot, and their elemental percentages are summarized in the bar chart for each composite separately. The percentages of the materials are roughly similarly distributed, which was the goal during the design and synthesis of the electrospinning solution. The highest occurrence of peaks was within the 2.5 keV level, so the plot was modified accordingly. Since some peaks overlap, it is not possible to visualize all of them, but it is possible to identify their location by their known value. Chromium in Cr-MOF@PVDF and iron in Fe-MOF@PVDF have intensities that are small and overlapped by oxygen and fluorine peaks, respectively. For Zn-MOF@PVDF, nitrogen, which is part of Zn-MOF, more precisely its ligand, was also detected; see description in Section 2.1. Compared to the other composites, the highest amount of metal was detected in the Zr-MOF@PVDF, namely 6.4% of zirconium.

### 3.2. Observation of Implemented MOFs in the Nanofibers

**Figure 5:**
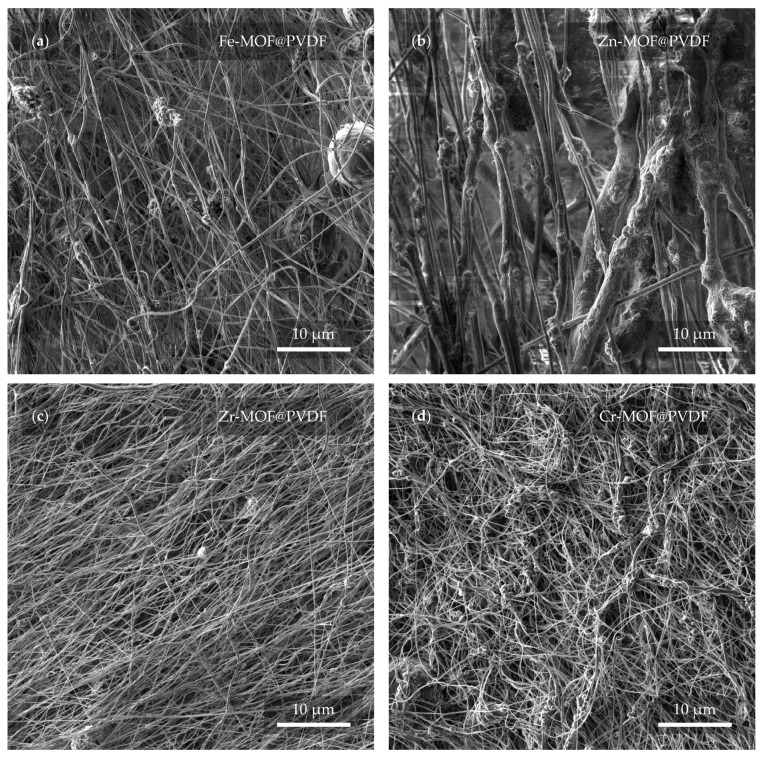
Preview of the membranes from a view field of 50 μm. In the micrographs, the membrane based on (**a**) iron, (**b**) zinc, (**c**) zirconium, and (**d**) chromium can be observed.

Electron microscope images in Figure 5 confirm that at first glance, all four materials are different in spite of the fact that the electrospinning parameters were not changed. It is therefore clear that the MOFs have an effect on the final shape and even the arrangement of the fibers.

The most distinguishing features of the studied samples are the zinc-based (Figure 5b) and zirconium-based (Figure 5c) fibers, where the diameter of the Zn-MOF@PVDF fibers is up to an order of magnitude larger than the Zr-MOF@PVDF fibers. It can also be observed from a larger scale that although the nanofibers should be relatively parallel to each other, the sample with Cr-MOF (Figure 5d), however, exhibits this condition the least, where fibers alignment cannot be confidently determined. Although in general, all samples are successfully spun and show no serious defects, imperfections in the Fe-MOF@PVDF sample (Figure 5a), such as spherical shapes of the balled-up polymer in the fibers, cannot be omitted. Droplet-like defects in the fibers, which are a common phenomenon, were not observed [32].

The observed differences in fiber diameter and the presence of balled-up polymer segments in Fe-MOF@PVDF were likely attributable to the influence of MOF type on the solution properties. Factors such as increased viscosity, heterogeneous dispersion, and uneven surface tension caused by Fe-MOF particles can lead to the formation of these bead-like defects. Additionally, insufficient solvent evaporation during electrospinning may contribute to the rounding of polymer at certain segments, resulting in spherical morphologies. Such effects were less prevalent in other MOF@PVDF composites, likely due to better compatibility or dispersion of those MOFs in the polymer matrix.

**Figure 6:**
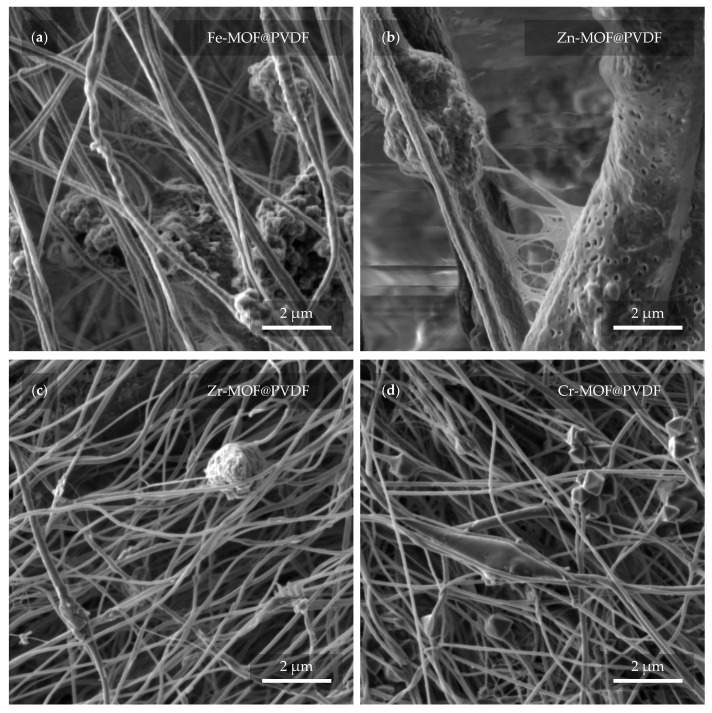
Detail of the nanofibers with MOF from a view field of 10 μm. In the micrographs, the PVDF with MOF based on (**a**) iron, (**b**) zinc, (**c**) zirconium, and (**d**) chromium can be observed.

A closer look at the capture and implementation of MOFs in fibers is provided in Figure 6. Several interesting details can be observed in different cases with varying coverage of MOFs. In Figure 6a, the Fe-MOFs have the shape of spheres that are attached to the fiber in a group of larger clusters. The diameter of the fibers usually does not exceed 1 μm. Zn-MOFs are trapped in similar clusters in Figure 6b. However, here, the fibers reach a multiply larger and more variable diameter in the range of hundreds of nanometers to units of micrometers. The porous structure of the individual fibers may prove interesting. It is also noticeable that there is a soft “web” between the fibers, which may be due to insufficient evaporation of the solvent during electrospinning. The evaporation of the solvent can be controlled by changing the parameters during the fabrication itself when the result is most predictable. However, the fibers can also be dried after spinning. In Figure 6c, the Zr-MOFs are trapped in small amounts, manifested by a slight diameter expansion of the fiber. Even here, a defect in the form of a balled-up polymer can be noticed. From the last image in Figure 6d, Cr-MOFs quite clearly resembles pyramidal structures with rectangular bases [33]. The thickness of the fibers is fairly comparable to the dimensions of Zr-MOF@PVDF. On closer examination, even the parallel orientation of the fibers, which was not obvious from Figure 5d, can already be distinguished.

**Figure 7:**
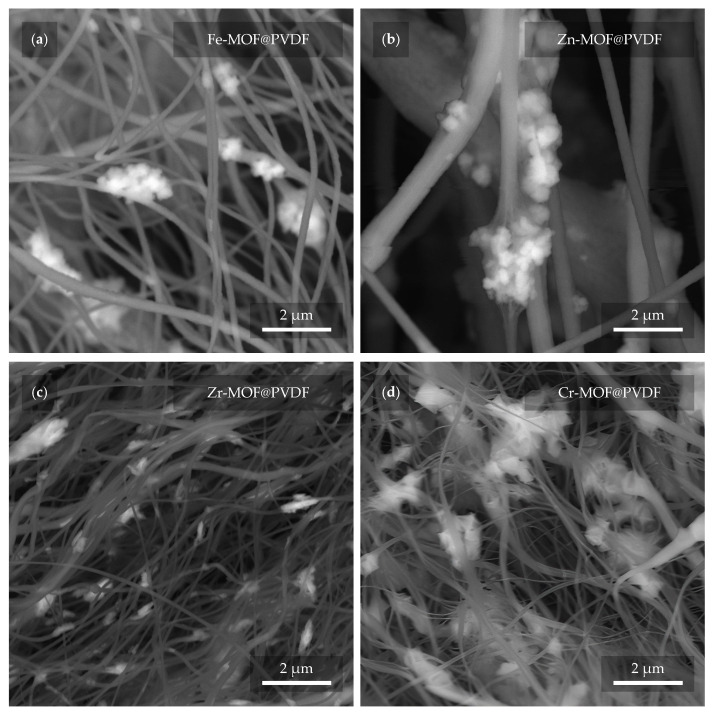
The MOFs (lighter parts) are distinguished from the PVDF fibers (darker parts) using a BSE detector. In the micrographs, the PVDF with MOF based on (**a**) iron, (**b**) zinc, (**c**) zirconium, and (**d**) chromium can be observed.

In the next close-up micrograph of the fibers (Figure 7), the MOFs are evident again, but this time confirmed by the BSE detector as brighter features in the image [34]. In this way, mistakes can be disproved where a MOF can be incorrectly detected as a defect in the fiber that may resemble it. At the same time, it is also possible to detect their actual presence and quantity due to the fact that a higher accelerating voltage was used in the configuration with the BSE detector (see Section 2.3). During such an observation, the fibers are more “transparent” to the depth of the material. In contrast, this feature is rather undesirable in the case of the previous observations. Therefore, for the other measurements, a much lower accelerating voltage of 2 kV was used. From the presented micrographs, Cr-MOFs show the highest material density (Figure 7d), whereas Zr-MOFs are found in smaller pieces (Figure 7c). Interestingly, however, as confirmed in the previous Figure 6, the fiber sizes between Zr-MOF@PVDF and Cr-MOF@PVDF are very similar.

### 3.3. Surface Contact Angle of Nanofibers with Liquid

**Figure 8:**
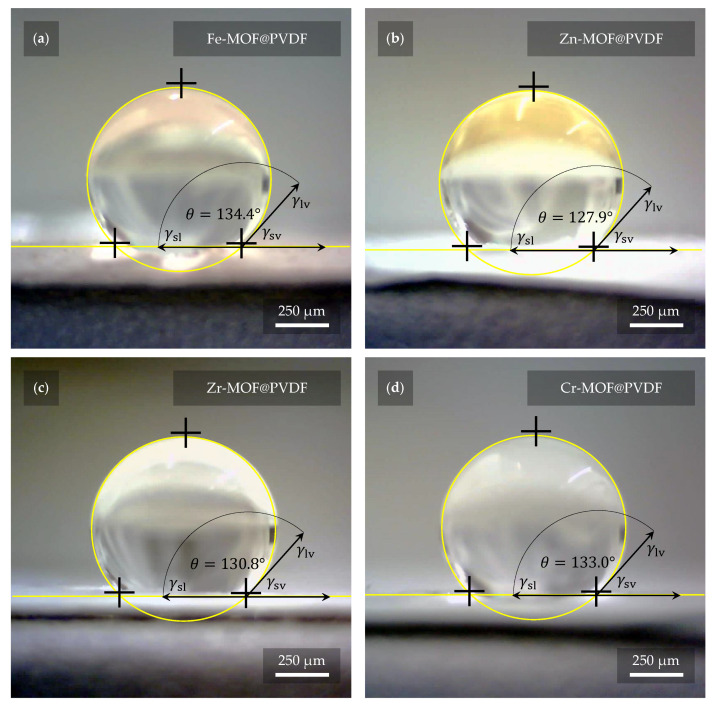
The highest hydrophobicity was measured with a value of 134.4° on sample (**a**) Fe-MOF@PVDF, while the lowest was measured on sample (**b**) Zn-MOF@PVDF with a value of 127.9°. Samples (**c**) Zr-MOF@PVDF and (**d**) Cr-MOF@PVDF had values of 130.8° and 133.0°, respectively. However, these are quite small differences, and thus it can be considered that the presence of MOF in the nanofibers does not contribute significantly to the different hydrophobicity of the fibers. The fibers without MOFs show 129.5° according to our measurements.

The degree of hydrophobicity was determined by measuring the contact angle of the liquid on all the fabricated samples. Interval values for hydrophilic materials range from 0° to 90° by standard. Interval values for hydrophobic materials range from 90° to 150°. If the range is exceeded even more, it is considered as superhydrophobicity. The observations from the measurements of these properties are represented in Figure 8. As the figure indicates, in all cases, the materials were evaluated as hydrophobic. More precisely, the mean contact angle was 134.4° for Fe-MOF@PVDF, 127.9° for Zn-MOF@PVDF, 130.8° for Zr-MOF@PVDF, and 133.0° for Cr-MOF@PVDF. As reference material, PVDF nanofibers without any dopant with identical fabrication parameters were also measured. Pure PVDF nanofibers showed a mean contact angle of 129.5°. Except for Zn-MOF@PVDF, the hydrophobicity of the composites used even increased compared to pure PVDF nanofibers. However, the resulting angles are very similar and within units of degrees, so it can be claimed that the deviations are minimal. Nevertheless, for specific applications such as filtration, more hydrophilic properties are desirable. The contact angle of the liquid with the material can be controlled in many ways. Oxygen plasma etching is quite effective, as reported by Havlíková T. et al. [35], where authors reduced the contact angle of PVDF nanofibers by up to 100°, with water soaking through the material after a few seconds. In our case, there was no visible water soaking even after one minute and the droplet was still stable on the material. Regarding plasma etching, it is advisable to choose parameters that do not completely disrupt the fibers and their structure. Another less invasive option is to spin the fibers in a different orientation, where they are not aligned in parallel [36,37,38]. However, as He Z. et al. [39] point out, this can reduce the value of the β-phase.

### 3.4. Sorption Performance of MOF@PVDF Membranes in Dye Removal Applications

A primary motivation for the incorporation of MOFs into PVDF membranes was to overcome the limitations of traditional polymer membranes in wastewater treatment, particularly in the removal of dyes such as MB, which are prevalent and persistent in industrial effluents. Pure PVDF membranes are known for their stability, but their adsorption capacity is limited due to the lack of chemically active binding sites.

**Figure 9:**
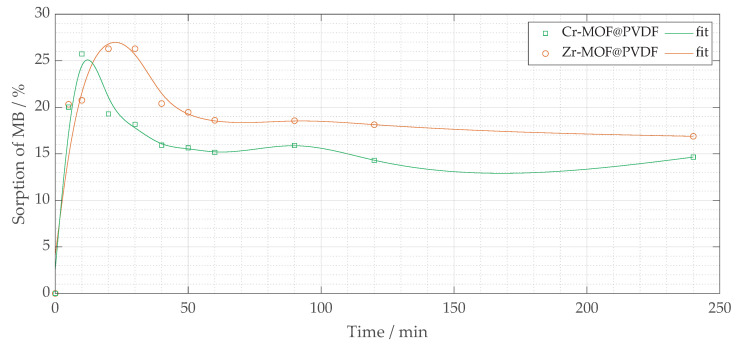
The percentage removal of methylene blue sorption (% MB) vs. *t* (minutes) for two fiber composites: Zr-MOF@PVDF and Cr-MOF@PVDF.

A preliminary investigation was conducted to assess the potential of these filters as photocatalysts for the degradation of organic dyes. Both Zr-MOF@PVDF and Cr-MOF@PVDF fibers were tested for MB, as shown in Figure 9. The results show that both composites exhibit rapid sorption of methylene blue within the initial contact period (0 to 50 min), primarily within the first 20 min, while the photocatalytic degradation process was not successful. This result is consistent with previous research in which active sites on the fiber surfaces readily bind MB.

Among the two composites, Cr-MOF@PVDF fibers demonstrated higher initial sorption efficiency, achieving approximately 25 to 27% MB removal within the first 50 min (mainly within 20 min), suggesting a higher affinity or greater availability of active sites compared to Zr-MOF@PVDF fibers. In contrast, Zr-MOF@PVDF fibers had a somewhat lower initial sorption efficiency, ranging from 20% to roughly 15%. After 150 min, both materials’ sorption capacities stabilized. Notably, Cr-MOF@PVDF fibers maintained a higher MB sorption percentage than Zr-MOF@PVDF, indicating a greater equilibrium sorption capacity. However, after 150 min, a slight fluctuation or decline in sorption was observed for Cr-MOF@PVDF, potentially due to the desorption of MB from the fiber surface or the limited stability of the Cr-MOF@PVDF composite under test conditions.

These findings suggest that the MOF@PVDF composites effectively adsorb methylene blue. The low removal percentage does not indicate a limitation, as the filters were tested in their unmodified form under a single sorption condition. In addition, their photocatalytic performance requires further optimization. Future research should focus on modifying the MOF composition, enhancing light absorption capabilities, and improving the stability of the composites to achieve efficient photocatalytic degradation of organic pollutants.

The sorption process observed in both Cr-MOF@PVDF and Zr-MOF@PVDF membranes suggests surface-mediated physisorption [31], likely governed by van der Waals forces [7], hydrogen bonding, and electrostatic interactions [22] between the dye molecules and MOF active sites [31]. The higher sorption rate within the initial 20 min is indicative of the abundance of readily accessible active sites [24]. Cr-MOF@PVDF’s sorption capacity (∼27%) compared to Zr-MOF@PVDF (∼20%) may be attributed to its higher surface roughness and porosity, as evident in SEM images (Figure 7). The Cr-MOF@PVDF’s structure, rich in carboxylate groups (confirmed by FTIR peaks at 1691 and ∼1402 cm^−1^), offers multiple interaction points for methylene blue through intermolecular forces. Additionally, while photocatalytic degradation was not observed, the adsorptive nature of these composites opens promising avenues for post-functionalization or UV-activation strategies to enhance photocatalysis. Future studies could investigate pH influence, isotherm modeling (Langmuir, Freundlich), and kinetic fitting (pseudo-first/second order) to fully characterize the sorption behavior. In practical wastewater treatment situations, it is important to assess factors like selectivity for different dyes, the potential for regeneration, and adsorption behavior under continuous flow conditions. However, the rapid absorption observed within the first few minutes highlights the membranes’ effectiveness for initial dye capture or pre-filtration processes.

### 3.5. Advancements over State-of-the-Art MOF@PVDF Composites

While previous research has demonstrated the potential of MOF@PVDF composites in various applications such as sensing, dye adsorption, or catalysis, many of these works are limited either by the exploration of only a single MOF type or by a narrow focus on a single property enhancement. In contrast, this study delivers a comparative, multi-MOF framework evaluating four chemically and structurally distinct MOFs (Fe-BTC, ZIF-8, UiO-66, and MIL-101(Cr)) integrated under identical synthesis and electrospinning parameters within a PVDF matrix. This allows for a direct comparison of how different MOF chemistries influence nanofiber formation, morphological characteristics, phase transitions, and functional properties such as wettability and sorption.

Beyond morphological observations, the study introduces a comprehensive multi-modal analysis, including FTIR for crystalline phase transitions, photoluminescence for electronic and defect structure evaluation, SEM with BSE/EDS for spatial and elemental mapping, and contact angle analysis for surface energy characterization. Such depth of characterization across MOF types in a unified matrix is rarely reported. For example, our photoluminescence data provide new insight into radiative recombination pathways, including metal–ligand charge transfer and defect–state emission phenomena largely overlooked in previous composite studies.

Notably, the findings highlighted that MOF-specific effects extended beyond structural characteristics: Zn-MOFs resulted in broader fiber diameter distributions and increased photoluminescence intensity due to surface defects and oxygen vacancies, while Cr-MOFs exhibited improved sorption efficiency and greater morphological uniformity, while Zr-MOFs exhibited lower emission but favorable nanostructure consistency. Results from contact angle measurements indicate that the inclusion of MOF did not compromise the hydrophobic properties of the PVDF membrane. This suggests strong compatibility and stability, which are critical for real-world applications, yet often under-discussed.

These insights enable a more informed approach to composite membrane design, where MOF selection is guided not just by chemical functionality, but also by its effect on morphology, optical response, and fluid interaction parameters often studied in isolation in the current literature. Therefore, this work contributes a new comparative framework for functional membrane development and provides empirical data to support future optimization of MOF@PVDF hybrids for environmental and sensing applications.

## 4. Conclusions

This work focused on the design, fabrication, and comprehensive characterization of nanofibrous membranes based on polyvinylidene fluoride (PVDF) integrated with metal–organic frameworks (MOFs) derived from iron, zinc, zirconium, and chromium. Using electrospinning, the uniform and defect-minimized membranes were produced under controlled parameters, and the successful incorporation of MOFs was verified through spectral, elemental, and microscopic analysis. Elemental distribution was found to be consistent across all composites, while surface contact angle measurements confirmed their high hydrophobicity, with only minor variation between the doped and pure PVDF fibers. FTIR and photoluminescence studies revealed structural differences linked to MOF type, including varying contributions to crystalline phase behavior and emission characteristics. Morphological evaluation showed that each MOF influenced fiber diameter and alignment differently, with Zn-MOF@PVDF demonstrating the greatest variability. Preliminary sorption testing on methylene blue solutions highlighted the rapid adsorption capacity of Cr-MOF@PVDF and Zr-MOF@PVDF membranes, with the former showing slightly higher removal efficiency. These results underline the potential of MOF@PVDF hybrid membranes for future development in dye removal, wastewater treatment, and functional filtration materials, while also providing insight into structure–property relationships that can guide further material optimization.

## Data Availability

The original contributions presented in this study are included in the article. Further inquiries can be directed to Nikola Papež. E-mail: papez@vut.cz.

## Abbreviations

The following abbreviations are used in this manuscript:

Ac	Acetone
BSE	Backscattered electrons
CV	Crystal violet
DMF	Dimethylformamide
EDS	Energy dispersive X-ray spectroscopy
FTIR	Fourier-transform infrared spectroscopy
LMCT	Ligand-to-metal charge transfer
IFE	Internal filtration effect
MB	Methylene blue
MLCT	Metal-to-ligand charge transfer
MOF	Metal–organic framework
PVDF	Polyvinylidene fluoride
P2VHR	Peak-to-valley height ratio
SE	Secondary electrons
SEM	Scanning electron microscopy
